# Identification and Anti-Fatigue Activity of Walnut Protein Hydrolysate

**DOI:** 10.3390/nu17061002

**Published:** 2025-03-12

**Authors:** Si Huang, Ya Wang, Manjia Li, Hongyu Mu, Chunlei Tan, Mingming Wang, Feng Zhang, Jun Sheng, Yang Tian, Cunchao Zhao

**Affiliations:** 1 College of Food Science and Technology, Yunnan Agricultural University, Kunming 650201, China; 18870918872@163.com (S.H.); wangya9188@126.com (Y.W.); muhongyu0710@163.com (H.M.); tcl98316@163.com (C.T.); wmm295813@163.com (M.W.); 15762196909@163.com (F.Z.); shengjunpuer@163.com (J.S.); tianyang1208@163.com (Y.T.); 2 College of Food Science and Engineering, Jiangxi Agricultural University, Nanchang 330045, China; 15979070480@163.com; 3 Engineering Research Center of Development, Utilization of Food and Drug Homologous Resources Ministry of Education, Yunnan Agricultural University, Kunming 650201, China; 4 Key Laboratory of Precision Nutrition, Personalized Food Manufacturing Ministry of Education, Yunnan Agricultural University, Kunming 650201, China; 5 Yunnan Research Center for Advanced Tea Processing, Pu’er University, Pu’er 665000, China; 6 Yunnan Plateau Characteristic Agricultural Industry Research Institute, Kunming 650201, China

**Keywords:** walnut meal, Dregea sinensis Hemsl. Protease, cecum microbiota, metabolomics

## Abstract

**Objective:** This study aims to investigate the structural and functional characteristics of walnut protein hydrolysates (WPHs) with different molecular weights prepared using protease from Dregea sinensis Hemsl, as well as the anti-fatigue effects of low-molecular-weight walnut protein hydrolysates (LWPs) and their impact on the cecal microbiota and faecal metabolism of mice. **Methods:** The anti-fatigue activity of WPHs with different molecular weights was evaluated, and the LWPs were analyzed in a centralized manner. A 28-day gavage study was conducted to assess LWP’s anti-fatigue benefits in mice, supplemented by metabolomic analysis to explore its impact on metabolic pathways. **Results:** Our findings revealed that LWP significantly outperformed unhydrolyzed walnut protein (WP) in terms of water retention, lipid retention, emulsifying properties, and foaming capacity. Notably, differential protein expression associated with LWP highlighted pathways related to antioxidant activity. In vivo studies showed that LWP markedly enhanced glycogen storage in the muscles and liver of mice, while reducing serum levels of serum urea nitrogen, lactate dehydrogenase, blood lactic acid, and creatine kinase. Furthermore, the levels of Superoxide Dismutase and Glutathione were significantly elevated, alongside a reduction in Malondialdehyde, indicating that LWP’s anti-fatigue effect is closely linked to improved oxidative stress resistance. Additionally, LWP promoted beneficial increases in microbial populations such as *Akkermansia*, *Alistipes*, *Eubacterium*, and *Muribaculum*, which are associated with enhanced fatigue resistance. Metabolomic analysis indicated significant enrichment in glycerophospholipid metabolism and amino acid biosynthesis, identifying key metabolites including palmitoylethanolamide and 4-methyl-5-thiazoleethanol, both of which are integral to health maintenance. **Conclusions:** LWP demonstrates a robust anti-fatigue effect, supported by its accessibility, straightforward preparation, and eco-friendly characteristics. These attributes suggest that LWP has promising potential for inclusion in health products aimed at enhancing vitality and combating fatigue.

## 1. Introduction

Fatigue refers to the inability of an organism to maintain its physiological functions at a certain level or the inability of the organism to maintain its due exercise intensity [1]. This can cause transient muscle or organ hypofunction [2], and usually includes three kinds of neurological, motor, and psychological fatigue [3]. If the fatigue state persists for more than 6 months, then it is called chronic fatigue [4]. The results of the survey conducted by the World Health Organization (WHO) show that the number of people who are in a fatigue state in the world accounts for more than 35% of the total number of people, of which nearly 60% are middle-aged men [5]. Along with the progress of modern society, the fast-paced life and great pressure of life have made fatigue-related syndromes a serious global health problem [3]; in addition, fatigue is also a complication of many diseases [6,7,8,9]. The pathophysiology and aetiology of fatigue have not yet been fully resolved, and the current types of medications for the relief of fatigue-related symptoms are limited and have side effects [10,11,12]; therefore, it is important to seek for functional foods with anti-fatigue effects instead of medications for the relief of fatigue-related interventions. In recent years, researchers have shown that natural active substances such as peptides, polysaccharides, amino acids, vitamins, polyphenols, alkaloids, etc., have the efficacy of relieving physical fatigue [13,14,15,16].

Walnuts are plants of the Juglans genus in the Juglandaceae family, which is rich in nutrients such as omega-3 and omega-6 unsaturated fatty acids, protein, fibre, vitamin E, B-complex, and minerals such as zinc, potassium, calcium, magnesium, and iron. These have the effects of reducing fat and benefitting energy levels, prolonging longevity, moistening the lungs, strengthening the kidneys, nourishing the blood, making hair healthy, and strengthening the brain, as well as preventing coronary heart disease [17,18]. According to relevant studies, walnut kernel contains rich bioactive components with important functions such as antitumour [19], antioxidant [20], anti-inflammatory, antimicrobial [21], insecticidal activity [22], and analgesic [23]. Walnuts are rich in protein, which is a macronutrient that assumes many human physiological functions [24]. At present, the human body can obtain proteins that can be utilised from animal, plant, and microbial sources [25]. From a nutritional standpoint, animal proteins have more nutrients than plant proteins but plant proteins are easier to digest [26], and from a resource point of view, the production of plant proteins is more economical and reasonable than animal proteins on the same area of land [27], which makes it clear that the study of plant proteins is of great significance. In addition, relevant studies have shown that adopting a plant-protein-based diet can reduce the risk of heart disease [28], diabetes, and certain cancers [29]. Walnut protein (WP) is a high-quality plant protein with economic, edible, and medicinal values [30], and its enzymatically produced bioactive peptides have unique physiological functions, such as anti-ageing [31], antioxidant [32], anti-radiation, fatigue elimination [33], and enhancement of the immune function of the human body. Anti-fatigue peptides [34] with easy absorption and few toxic side effects are of great research significance in the fields of food and medicine.

Dregea sinensis Hemsl. Protease, derived from the perennial climbing woody vine Dregea sinensis, is a novel enzyme system with significant industrial potential. Traditionally, aqueous extracts from its stems have been utilized as a natural coagulant in Yunnan, China, for over six centuries to produce milk pastry (a traditional milk cake), replacing conventional acid-based curdling agents. Recent biochemical characterization reveals that this protease complex predominantly consists of a cysteine protease, procerain B, with a molecular weight of 23.8 kDa, exhibiting exceptional thermostability and pH tolerance [35]. These properties underscore its dual role as a sustainable alternative in dairy processing and a promising biocatalyst for functional food development.

In this study, we aimed to investigate whether low-molecular-weight walnut protein hydrolysates (LWPs), prepared through enzymatic digestion of walnut proteins using Dregea sinensis Hemsl. protease, can alleviate exercise-induced fatigue in mice. Specifically, we explored the effects of LWP on fatigue using a weight-bearing exhaustion swimming test, while also examining its impact on the intestinal microbiota and metabolic profiles of mice. The findings of this study are intended to provide scientific evidence for the development of functional foods targeting fatigue management as an adjunct to clinical practice.

## 2. Materials and Methods

### 2.1. Materials and Reagents

Walnut protein was extracted from walnut meal and industrially produced. The Dregea sinensis Hemsl was harvested from the Kunming hills by experienced farmers, and we extracted the Dregea sinensis Hemsl Protease by graded chromatography. Blood urea nitrogen (BUN), lactate (LD), creatine kinase (CK), hepatic glycogen, and myo-glycogen assay kits were purchased from Nanjing Jianjian Institute of Biotechnology (Nanjing, China). Bicinchoninic acid (BCA) protein concentration kit, ECL reagent, and methanol were purchased from Biyuntian Institute of Biotechnology (Shanghai, China); C_6_H_8_O_7_, NaOH, HCl, and (NH_4_)_2_SO_4_ from Chengdu Jinshan Chemical Reagent Co., Ltd. (Chengdu, China).

### 2.2. Preparation of WPHs

Referring to the method of Jin [36] with modification, walnut protein was mixed with ultrapure water at a material–liquid ratio of 1:10 and then placed at 60 °C for 12 min of sonication. The pH was adjusted to 8.6 using 1 mol/L NaOH and Dregea sinensis Hemsl. Protease with a substrate concentration of 3% was added, and the enzymatic hydrolysis was carried out for 4.5 h in a constant-temperature water bath. Following, the enzymes were inactivated for 10 min at 90 °C after the end of the enzymatic hydrolysis, and then lowered to room temperature and centrifuged for 15 min in a low-temperature centrifuge at 4000 r/min. Then, it was reduced to room temperature, and the hydrolysate obtained was centrifuged in a low-temperature centrifuge at 4000 r/min for 15 min. The supernatant, i.e., WPHs, was taken, and a portion of it was removed and lyophilised for spare use, the remaining was subjected to ultrafiltration.

### 2.3. Ultrafiltration Crude Separation

The WPHs were purified by the ultrafiltration membrane method to isolate walnut proteolytic proteins with molecular weights less than 3000 Da (LWPs), walnut proteolytic proteins with molecular weights between 3000 and 5000 Da (MWPs), and walnut proteolytic proteins with molecular weights greater than 5000 Da (HWPs).

### 2.4. Functional Characterisation of WPHs

A total of 200 mg of sample (*m*_1_) was added to the centrifuge tube and the weight of the tube was recorded (*m*_0_). Then, 5 mL of water or corn oil was added to the tube, vortexed continuously for 1.5 h. The tube was centrifuged at 5000× *g* for 10 min and the supernatant was removed and weighed (*m*_2_). The water-holding capacity (WHC) and oil-holding capacity (OHC) were calculated as follows:

$$WHC\left(g/g\right)=\frac{m_{2}-m_{1}-m_{0}}{m_{1}}$$

$$OCH\left(g/g\right)=\frac{m_{2}-m_{1}-m_{0}}{m_{1}}$$

The samples were weighed in 100 mL of distilled water, prepared into neutral protein peptide solutions of different concentrations, and the initial foam volume (*V*_0_) was recorded after 10 min at different temperatures. Homogenisation was performed in a high-speed tissue masher at 10,000 r/min for 3 min, the foam volume (*V*_1_) was accurately recorded at the time of stopping of homogenisation, and the foaming stability of the samples was calculated according to the following formula (*FC*). The foam volume (*V*_2_) at the end of homogenisation for 30 min was recorded at room temperature, and the foaming stability (*FS*) of the samples was calculated according to the formula. Each sample was measured 3 times:

$$FC=\frac{V_{1}}{100}\times 100\%$$

$$FS=\frac{V_{2}}{V_{0}}\times 100\%$$

The emulsifying ability index (*EAI*) and emulsion stability index (*ESI*) of the samples were determined by dissolving the samples in PBS solution (10 mM, pH 7.2) to form a 10 mg/mL dispersion. Then, 15 mL of the peptide solution was mixed with 5 mL of corn oil and homogenised for 2 min. The homogenised emulsion (0.5 mL) was diluted with 50 mL of 0.1% SDS and the absorbance was measured at 500 nm (*A*_0_). The diluted emulsion was detected under the same conditions after 10 min and was recorded as *A*_1_. The 0.1% SDS absorbance was used as a blank control. The results were calculated by the following formula:

$$EAI\left(m^{2}/g\right)=\frac{2\times 2.303\times A_{0}\times DF}{C\times \emptyset \times \theta \times 10000}$$

$$ESI=\frac{A_{0}\times 10}{\Delta A}$$

where *DF* is the dilution factor; *C* is the protein concentration; and the oil volume fraction, 0.01 m [optical range], is the difference between *A*_0_ and *A*_1_.

### 2.5. Scanning Electron Microscope Analysis (SEM)

A total of 1 mg of each sample powder was taken and uniformly coated on a specimen holder adhered with double-sided conductive tape, sprayed with gold coating treatment for 5 min to ensure that the samples were encapsulated by gold, and then observed and photographed using a scanning electron microscope, for which the accelerating voltage was 15.0 kV, the working distance was 12.8 mm, the magnification was 20,000×, and the working temperature was 25 °C.

### 2.6. X-Ray Diffraction (XRD)

A total of 10 mg of each sample powder was taken and ground uniformly. Then, the samples were loaded into a sample plate, placed in a holder, and X-ray diffraction data were collected using an XRD diffractometer (Shanghai Aiyitong Network Technology Co., Ltd., Shanghai, China).

### 2.7. Fourier-Transform Infrared Spectroscopic Analysis (FTIR)

In total, 1 mg of each sample powder was taken and mixed with 100 mg of dried KBr, uniformly ground to less than 2.5 μm, and pressed using the KBr pressing method. The samples were scanned using FTIR over a scanning range of 4000 to 500 cm^−1^, with 16 sweeps, and a spectral resolution of 4 cm^−1^, therefore, the absorption spectra were recorded.

### 2.8. Peptide Identification

Based on the preliminary pre-tests, it was shown that the anti-fatigue activity of LWPs with a molecular weight less than 1000 Da was slightly better than that of LWPs with a molecular weight less than 3000 Da. Since we are targeting health food products, it is more economical to choose an LWP with a molecular weight of less than 3000 Da, which can greatly reduce the R&D cost given the small difference in anti-fatigue activity. LWP samples with 0.2 μg molecular weight less than 3000 Da were taken and separated by a nano-UPLC liquid-phase system and then coupled to a mass spectrometer equipped with a nanolitre ion source for data acquisition. The chromatographic separation was performed on a 75 μm ID × 15 cm reversed-phase column, and the mobile phases were acetonitrile–water–formic acid, with 0.1% formic acid–water in mobile phase A and 0.1% formic acid–acetonitrile in mobile phase B. The raw data were collected using a SpectroMicroSystems^®^ liquid-phase system. The raw data files were searched using SpectroMine software (Tencent cloud, https://biognosys.com/software/spectromine/ (accessed on 7 March 2025)) with the Pulsar search engine, and qualitative analyses were carried out at the end of the search.

### 2.9. Animal Experimental Design

All animals were kept in accordance with the guidelines for experimental animals, and the animal experiments were approved by the Institutional Animal Care and Utilisation Committee of Yunnan Agricultural University. Eighty 6-week-old male ICR mice of 18–22 g were selected and kept in a clean room for experimental animals with cool ventilation, 50–60% relative humidity, and a room temperature of (25 ± 2) °C, and the mice were free to eat and drink during the feeding period. After one week of adaptive feeding, the mice were screened for swimming, and mice that could not swim or had uncoordinated swimming postures were excluded. Seventy-two mice were finally selected. The eligible mice were randomly divided into 6 groups: group A—quiet control group (q-con); group B—exercise model group (e-con); group C—positive vitamin C control group (100 mg/kg PV); group D—walnut proteins peptide low-dose group (50 mg/kg LWP-L); group E—walnut proteins peptide medium-dose group (100 mg/kg LWP-M); Group F—high-dose group of walnut protein peptide (200 mg/kg LWP-H). Dose selection was based on pre-experimentation. Twelve mice in each group—the control and model groups—were gavaged with the corresponding dose of saline every day, the experimental cycle was 28 d, and the mice in each group were weighed every day.

After the last gavage for 30 min, six mice were randomly removed from each group. The mice were given 10% of their body weight in lead, and placed in a swimming box at a water depth of 40 cm and a water temperature of 25 °C. The time from the start of swimming to the time when the head was completely submerged in the water and could not rise to the surface for 8 s was recorded as the time of the weight-bearing swimming of the mice. The remaining six mice in each group were dissected.

### 2.10. Biochemical Analysis of Serum and Liver, Muscle

Biochemical analyses of serum and liver and muscle samples were performed, including hepatic glycogen (HG), myo-glycogen (MG), serum urea nitrogen (BUN), blood lactate (LA), creatine kinase (CK), and lactate dehydrogenase (LDH). Muscle tissues were fixed with 4% paraformaldehyde, embedded in paraffin, sectioned and stained with hematoxylin and eosin (H&E), followed by light microscopy for routine morphological analysis.

### 2.11. Gut Microbiota Analysis of Cecum Contents

Bacterial DNA was isolated from cecum contents and prepared for the 16S rRNA gene sequencing analysis. The 16S rRNA gene was subjected to sequencing of the V3-V4 region and data processing, and the 16S rRNA gene was amplified with forward primer (50-CCTACGGRRBGCASCAGK VRVGAAT-30) and reverse primer (50-GGACTACNVGGGTWTCTAATCC-30) to amplify the V3-V4 region of the 16S rRNA gene.

### 2.12. Faecal Metabolomics Analysis

Sample handling methods, NMR data acquisition, and processing were performed using previously described methods. Raw data were preprocessed using MestReNova 10.0 and multivariate analyses were performed using SIMCA 14.0 software (Umetrics, ume, Sweden), including principal component analysis (PCA) and orthogonal partial least-squares discriminant analysis (OPLS-DA). In OPLS-DA, potential biomarkers that contributed significantly to group separation were identified based on variable importance projection (VIP) values > 2 and *p* < 0.05. Metabolomics pathway analysis (MetPA) was performed using MetaboAnalyst (McGill University, Montréal, QC, Canada, https://www.metaboanalyst.ca/).

### 2.13. Statistical Analyses

All data are expressed as mean ± SD, the statistical differences were analysed by ANOVA and Tukey’s test, and the normality of the data was tested prior to ANOVA and post hoc tests. Data were analysed using SPSS 24.0 (SPSS Inc., Chicago, IL, USA), GraphPad Prism version 5.01 (GraphPad software, San Diego, CA, USA), and correlations were analysed by Spearman’s correlation. Heatmaps were created using the Heml package (Heatmap Illustrator, version 1.0). *p*-values < 0.05 were considered statistically significant differences.

## 3. Results

### 3.1. Functional Properties of WPHs

**Figure 1:**
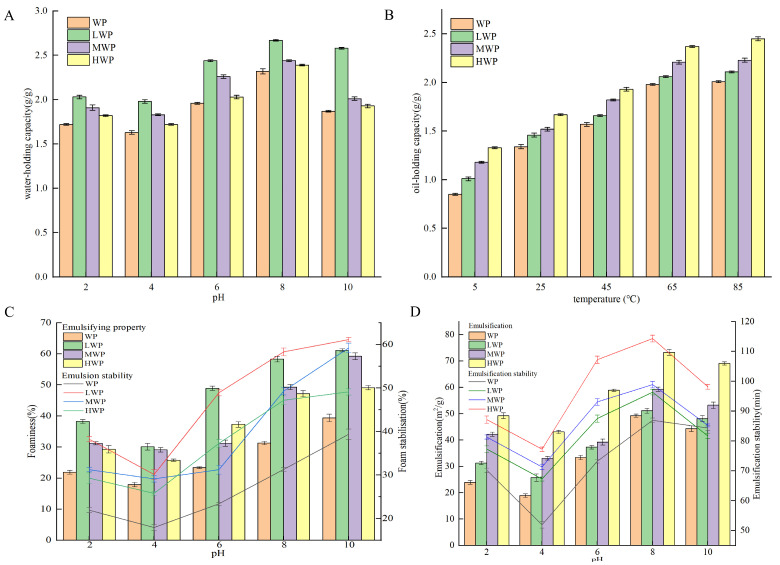
(**A**) Water-holding capacity, (**B**) oil-holding capacity, (**C**) emulsification stability, and (**D**) foaming stability of WP and WPHs of various molecular weights.

Water-holding capacity is the ability to capture and immobilise water after the formation of water-based colloids under external forces and is an important property of proteins in the food industry [37]. Figure 1A shows that the water-holding capacity of walnut proteins was significantly increased by enzymatic hydrolysis, which may be related to the high solubility of the enzymatic products. The WPHs were more easily dispersed in water, which led to a decrease in the free water and an increase in the bound water, which resulted in an increase in the water-holding capacity—with the best water-holding capacity of the LWPs—which may be due to the fact that their small-molecule peptide chains contain more hydrophilic groups. The water-holding capacity of the WPHs was the lowest at a pH of 4, and with an increase in pH the water-holding, capacity increased. The water-holding property increased probably due to the fact that WPHs exist in ionic form away from the isoelectric point, which enhances the solubility and viscosity of the protein [38]. Oil-holding capacity affects the organoleptic quality of protein products and their physical properties during food processing and storage. Figure 1B shows that the oil-holding capacity of WP was significantly increased by enzymatic digestion and continued to increase over the range of measured temperatures, probably due to the increase in temperature that increased the lipophilic binding sites in the side chains of amino acid residues of WPHs. The oil-holding capacity of the WPHs stabilised at 65 °C and reached a maximum value of 2.39 g/g at 85 °C. Emulsification refers to the ability of proteins to form emulsions at the oil–water interface, whereas the emulsification stability measures the ability of the emulsion to maintain its structure and state [39]. Figure 1C shows that the enzymatic treatment enhanced the emulsifying ability of WPHs, probably due to their increased solubility, which promoted the migration to form a cohesive film at the water–oil interface and improved the emulsibility. A pH of 4 showed the lowest emulsifiability and emulsion stability of the WP and WPHs, and deviation from the isoelectric point resulted in the enhanced solubility and dispersion of proteins, which promoted the stability of the oil–water interface. Foaming properties reflect the ability of proteins to migrate to the air–water interface and form a rigid film [40]. Figure 1D shows that the foaming property and foam stability of WP were enhanced at different pH values after enzymatic digestion, probably due to the stretching of the peptide chains of WPHs that reduced the surface tension of the liquid bubbles and enhanced the bubble stability. A pH of 4 showed the lowest foaming property and foam stability of the WP and WPHs, which increased gradually when deviating from the isoelectric point and reached the maximum value at pH 8. In addition, the foaming property and foam stability of HWP were better than those of LWP and MWP.

### 3.2. SEM, XRD, and FTIR Analyses

**Figure 2:**
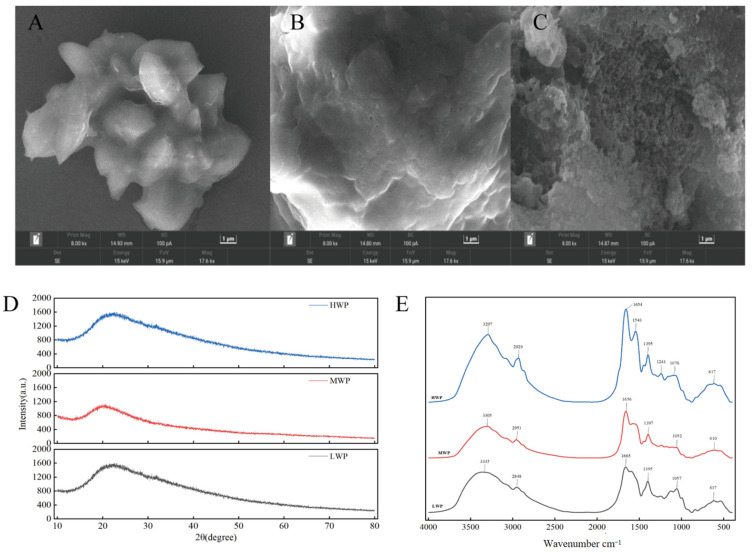
(**A**) SEM of LWP, (**B**) SEM of MWP, (**C**) SEM of HWP, (**D**) XRD plots of WPHs with different molecular weights, and (**E**) FTIR plots of WPHs with different molecular weights.

In order to explore the surface microstructure of each molecular segment of walnut protein digests, scanning electron microscopy was used. As shown in Figure 2A, the surface of low-molecular-weight walnut peptide (LWP) appears smooth. In contrast, the microscopic surface of medium-molecular-weight walnut peptide (MWP), shown in Figure 2B, shows a fish-scale-like structure and a less-smooth surface than that of LWP. Figure 2C demonstrates high-molecular-weight walnut peptide (HWP), which has a lamellar or blocky surface and a large, more porous, and sparse surface. These observations suggest that there are significant differences in the surface microstructure of walnut peptides with different molecular weights, and that the surface of walnut peptides with smaller molecular weights is relatively smoother.

X-ray diffraction is a good analytical method to study the microstructure of substances by crystallographic changes. From the diffraction patterns of MWP and HWP shown in Figure 2D, a major weakly dispersed diffraction peak is observed near 2θ of 20°, while LWP has a weakly dispersed diffraction peak near 2θ of 22.4°, which suggests that LWP, MWP, and HWP are not in an ordered arrangement but are randomly amorphous structures. All three have diffraction peaks near 2θ of 20°, presumably due to the α-helical structure in LWP, MWP, and HWP.

IR spectra are usually used to detect the formation and composition of organic functional groups, mainly including O-H, C-O, and N-H. The bands between 4000 cm^−1^ and 500 cm^−1^ were selected and deconvolved after baseline correction. As can be seen in Figure 2E, there is a high-frequency absorption peak at around 3300 cm^−1^ for LWP, MWP, and HWP, which is caused by the O-H and N-H stretching vibrations, while 2929 cm^−1^, 2951 cm^−1^, and 2848 cm^−1^ correspond to the C-H stretching vibrations. In addition, amide I band vibrations caused by C=C stretching were observed at 1654^−1^, 1656^−1^, and 1665^−1^, and amide III band vibrations caused by C-H bending at 1359^−1^, 1397^−1^, and 1395^−1^. The presence of similar bands for different molecular weights of walnut protein digests indicates the presence of the same major functional groups for LWP, MWP, and HWP.

### 3.3. Identification and Screening of LWP

**Figure 3:**
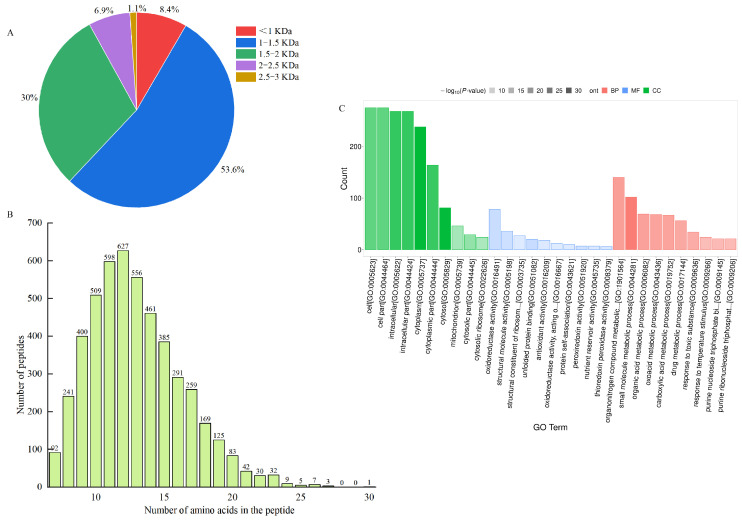
(**A**) Molecular weight distribution of peptides; (**B**) chain length peptide identification of peptides; (**C**) GO functional annotation.

Ultrafiltration can intercept large molecules and effectively separate and enrich low-molecular-mass peptides with high biological activity [41], which can also reveal the mechanism of LWP activity at the molecular structure level [42]. In this study, WPHs with different molecular masses were prepared by ultrafiltration separation. In our anti-fatigue pre-tests, WPHs with molecular weights less than 3000 Da were found to have significant anti-fatigue effects. Therefore, we identified the amino acid sequences of LWPs with molecular weights less than 3000 Da by LC-MS/MS, and 2927 peptide sequences were identified from LWPs by uniprot database comparison analysis. As shown in Figure 3A,B, these peptides mainly consisted of 7−20 amino acids, and were dominated by short peptides with molecular masses less than 1500 Da (61%).

**Figure 4:**
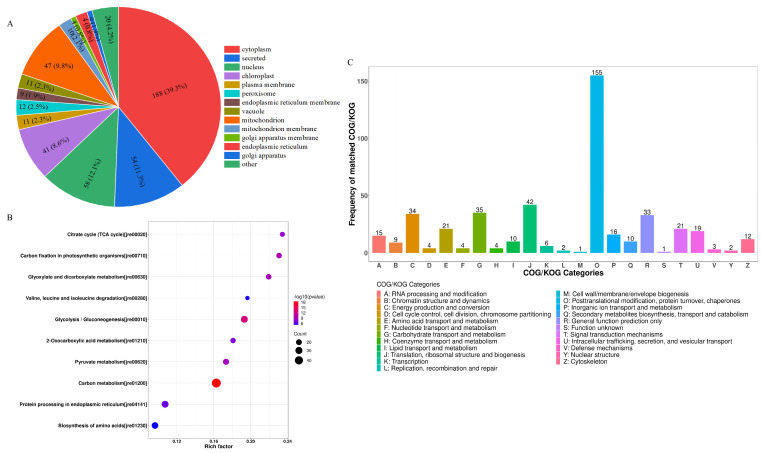
(**A**) Subcellular localisation; (**B**) KEGG analysis; (**C**) COG functional classification.

The differentially expressed LWPs were subjected to GO functional annotation, Figure 3C, to determine the biological functions they mainly exercise, and a total of 30 significant GO entries were annotated for the differentially expressed proteins. Among them, in the cell component, differentially expressed LWP was mainly involved in the cell (GO:0005623), cell part (GO:0044464), intracellular (GO:0005622), and cytoplasm (GO:0005737); in molecular function, differentially expressed LWP was mainly localised in oxidoreductase activity (GO:0016491), structural molecule activity (GO:0005198), and unfolded protein binding (GO:0051082), etc.; in biology processes, differential expression of LWP in the organonitrogen compound metabolic process (GO:1901564), small molecule metabolic process (GO:0044281), and organic acid metabolic process (GO:0006082) were significantly enriched. Thus, the main differential expression of LWP was in oxidoreductase activity and molecular metabolism, and had significant effects on cellular and intracellular components. KEGG is an informational network connecting known molecules to each other, and the top 10 most significantly enriched classified pathways, Figure 4B, shows that the differentially expressed proteins were annotated to 32 KEGG signalling pathways in the KEGG database, which were mainly involved in the carbon metabolism pathway, metabolic pathways pathway, biosynthesis of secondary metabolites signalling pathway, glycolysis/gluconeogenesis pathway, protein processing in the endoplasmic reticulum pathway, and the ribosome pathway. The results of database comparisons, Figure 4C, show posttranslational modification, protein turnover, chaperones, carbohydrate transport and metabolism, energy production and conversion, amino acid transport and metabolism, and signal transduction mechanisms. Among them, the most frequently occurring proteins were cellular process and signal transduction proteins, and the least frequently occurring proteins were information storage and processing proteins; the function of one protein was undetermined. Subcellular structure annotation of LWP differentially expressed proteins was performed using PSORTb software (https://www.psort.org/psortb/ (accessed on 7 March 2025)), and the results in Figure 4A show that 466 differentially expressed proteins were localised to 17 entries, which were 188 cytoplasm, 54 secreted, 58 nucleus, 47 mitochondrion, 41 chloroplast, 12 peroxisome, 11 vacuole, 11 plasma membrane, 10 mitochondrion membrane, 9 endoplasmic reticulum membrane, 9 endoplasmic reticulum, 4 Golgi apparatus membrane, 4 Golgi apparatus, 3 chloroplast membrane, 3 vacuole membrane, 1 peroxisome membrane, and 1 plastid. Taken together, the identification results all indicate that LWP possesses antioxidant function, which echoes the results of antioxidant indices in animal experiments.

### 3.4. LWP Reduces Fatigue Induced by Strenuous Exercise

**Figure 5:**
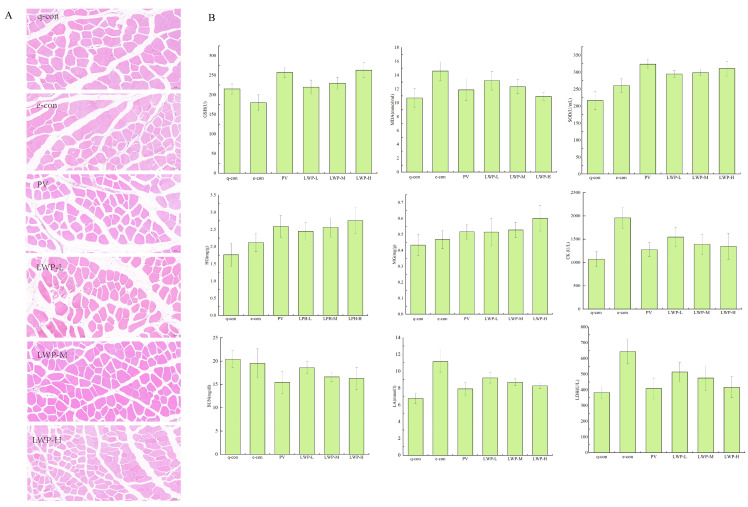
(**A**) Fatigue indicators; (**B**) muscle morphological analysis of mice in each group at 20× magnification.

Muscle damage can be visualised by looking at the morphology of the muscle tissue. Figure 5A shows the results of the HE staining of muscle fibres. As shown in the figure, the skeletal muscle cells of the quiet control group and the exercise control group were complete and neatly arranged; the fibroblasts of the positive control group with gavage of Vc were neatly arranged and full, with complete morphology and hypertrophied myofibres. Myofibroblasts in the walnut protein peptide group were morphologically complete and myofibrils were hypertrophied. The number of myosatellite cells was significantly reduced compared with the two control groups. This suggests that the walnut protein peptide intervention has a protective effect on skeletal muscle, reduces the damage to skeletal muscle cells caused by exercise, and enhances the exercise capacity of mice.

As shown in Figure 5B, LWP prolonged the weight-bearing swimming time of mice by 6.36–28.91% compared with the exercise control group. In terms of glycogen consumption, the hepatic glycogen (HG) content of mice in the LWP-H group was 15.39–30.08% higher than that of the exercise control group, and it also significantly reduced the consumption of myo-glycogen (MG) in mice; moreover, LWP cleared the accumulation of lactic acid (LA) and serum urea nitrogen (BUN) in the blood of the mice (*p* < 0.05), and the LWP group’s blood LA and BUN levels were reduced by 17.42–25.92% and 4.8–16.61%, respectively, compared with those of the exercise control group. LWP was able to significantly (*p* < 0.05) reduce serum creatine kinase (CK) and lactate dehydrogenase (LDH) activities in rats, which resulted in the reduction in CK’s viability in serum by 21.04–31.23% and LDH’s viability in serum by 20.27–35.23% compared with that of the exercise control group. LWP was also capable of reducing the accumulation of urea nitrogen (BUN) in the blood of mice in the LWP-H group, which decreased by 35.35%; therefore, our extracted LWP has better anti-fatigue efficacy. LWP can effectively increase the activity of antioxidant enzymes GSH and SOD in the serum of mice (*p* < 0.05), and decrease the content of MDA in serum (*p* < 0.05) so we judge that the anti-fatigue process may be related to the enhancement of its oxidative stress capacity. In the identification of LWP, this molecular weight peptide was found to have a significant antioxidant signalling pathway, and the results were consistent with oxidative indicators.

### 3.5. Effect of LWP on Intestinal Flora of High-Intensity Exercise Mice

**Figure 6:**
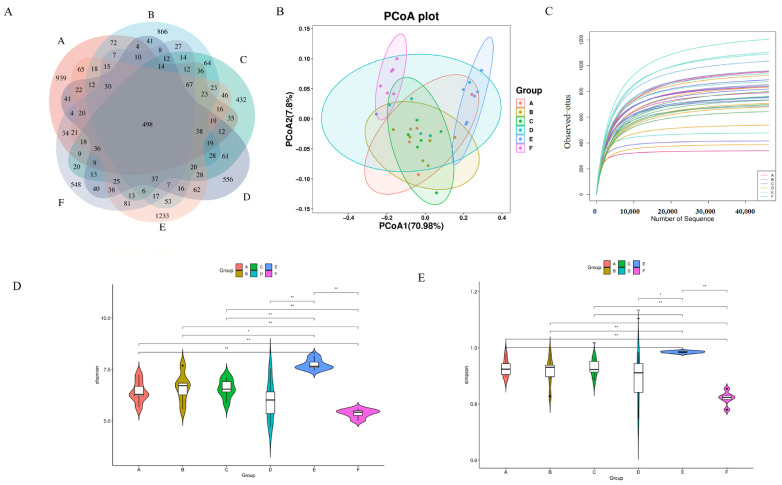
(**A**) Wayne plot of ASVs (Groups A–F are subgroups for animal experiments, as described in 2.9); (**B**) PCoA plot; (**C**) dilution curve; (**D**) Simpson’s exponent; (**E**) Shannon’s exponent. * *p* ≤ 0.05, ** *p* ≤ 0.01.

The feature sequences obtained after quality control and denoising are represented by Venn diagrams showing the number of unique ASVs and shared ASVs in each group. As shown in Figure 6A, the e-CON, q-CON, VC, LWP-L, LWP-M, and LWP-H groups contained 2174, 2114, 1703, 1780, 2623, and 1710 ASVs, respectively, and the number of total ASVs in the six groups was 498. pCoA analysis showed that there was a significant difference between the LWP-H group and the q-con, with 70.98% for PC1 and 7.8% for PC2, see Figure 6B. The dilution curves indicated that the amount of sequencing data could reflect the species diversity of the samples, and the curves all tended to be flat, which could cover most of the samples to a certain extent, as seen in Figure 6C. α diversity is an indicator of species richness, diversity, and homogeneity, and is also known as diversity within habitats. Studies have shown that the consumption of certain functional foods or ingredients can modulate the composition and diversity of the intestinal microflora, and whether LWP—as a bioactive ingredient from walnut proteins—may also exert certain effects by modulating intestinal dysbiosis in C57 mice, for which intestinal contents of mice were collected for 16S rRNA gene sequencing. The results of the α-diversity analysis of the intestinal flora, shown in Figure 6D,E, displays that the diversity indices of the intestinal flora of mice in the LWP-L group, Simpson’s index, and Shannon’s index were significantly higher than those of the other five groups, which indicated that the number of microbial community species and homogeneity were high in the LWP-L group. Compared with the blank group, the abundance, coverage, homogeneity, and diversity of the walnut peptide group changed—in which the diversity index changed significantly, indicating that the structural composition of the intestinal flora changed. Among the three experimental groups, the LWP group had the highest abundance and diversity indices, which were significantly higher than those of the LWP-M and LWP-H groups, suggesting that LWP altered the structural composition of the intestinal microbiota of mice but more significantly in the low-dose group.

### 3.6. Structural Composition of Microbial Community

**Figure 7:**
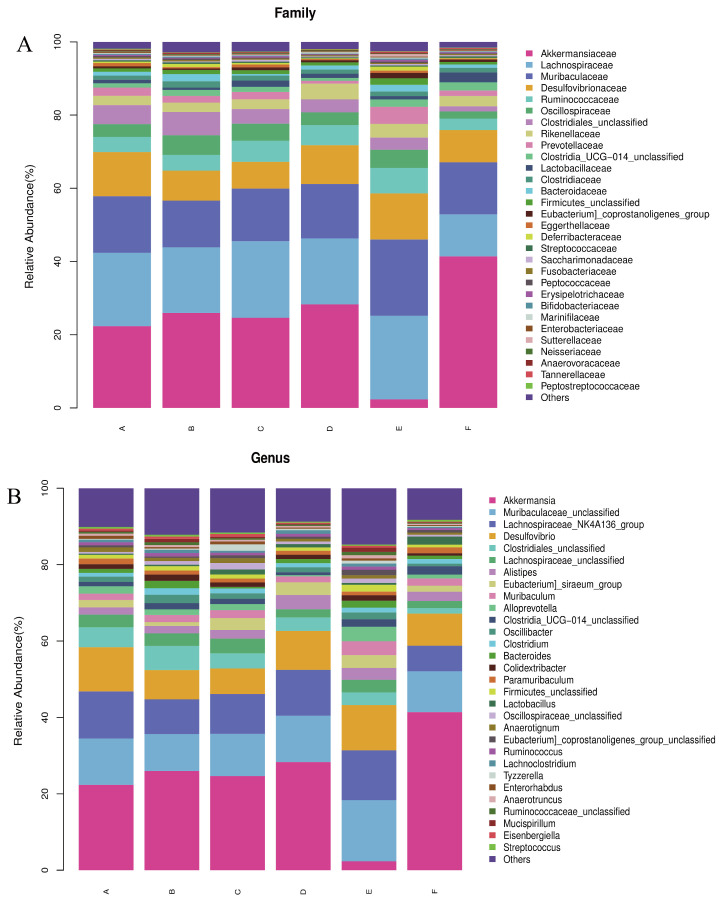
(**A**) Relative abundance of bacteria at the phylum level; (**B**) relative abundance of bacteria at the genus level. (Groups A–F are subgroups for animal experiments, as described in 2.9).

Based on the results of the assay, the relative abundance of bacteria at the phylum and genus levels was plotted. As shown in Figure 7A,B, at the phylum level, the most prevalent bacteria in each group were *Thick-walled bacteria*, *Warble microbacteria*, *Bacteroidetes*, and *Desulfovibrio*. At the genus level, *Thick-walled bacteria*, *Warty microbacteria*, *Bacillus mimics*, and *Desulfurised bacteria* were prevalent in the six taxa. At the phylum level, relative to q-con, an increase from 40.26% to 44.13% was observed in the *Thick-walled phylum* of PV, an increase of 8.32% in the *Thick-walled phylum*, and a decrease of 20% in the *Warty microflora* in the LWP-M group, and there was no dose-dependence between the alteration of the bacteria at the phylum level and the subjects. Whereas the LWP-H group had an increase of 19.06% in the *Wart microflora gates*, the *Anamorphic bacillus* gates were almost the same as the rest of the groups, whereas the LWP-M group had an increase of 10%. The number of *Desulfovibrio* spp. in the three experimental groups did not differ much between the control and positive groups. At the genus level, the genus *Micrococcus wartyi* was the most abundant in LWP-L and LWP-H compared to q-con, with an increase in abundance in both experimental groups (5.99 per cent in LWP-L and 19.06 per cent in LWP-H). The relative abundance of *Muribaculaceae* was highest in the LWP-M group, and decreased by 0.02 per cent in the LWP-L group and by 1.5%.

**Figure 8:**
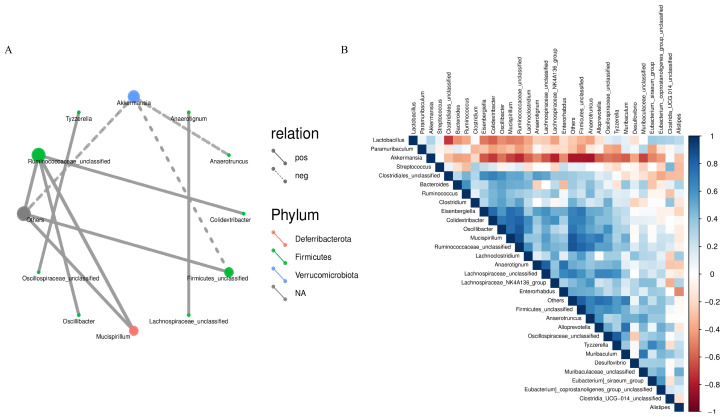
(**A**) Correlation network diagram of mouse gut microbial communities; (**B**) OTU-based heatmap of correlation of mouse gut flora.

In the microecological study, we also compared the association analyses between species and environmental factors. The correlation between species was calculated by the abundance and change relationship of different species in each sample, and the species that were correlated with each other were found by filtering under certain conditions. We defaulted to show the relationship pairs with correlation coefficient |rho| > 0.8, as shown in Figure 8A, the gut microbial communities of mice gavaged with LWP all formed a unique microbial network containing 12 nodes and 11 edges, with a positive correlation of 72.73% and a negative correlation of 27.27%, and the nodes of their network mainly belonged to the phylum *Thick-walled Bacteria* and the warty microbial phylum, which accounted for 90.9% of all the nodes and were the dominant phylum of the bacterial community. To further investigate the effect of genus level LWP on the level of enteric bacteria, we plotted a heatmap at the genus level, and as can be seen in Figure 8B, the remaining groups showed changes in other strains with lower abundance compared to q-con, and all of them showed a positive correlation with AKK bacteria. In the e-con group, strain abundance increased in *Clostridium* and *Bacteroides* and decreased in Anaerotignum. In the LWP-M group, the increase was in *Alistipes*, *Eubacterium siraeum*, and *Muribaculum*.

### 3.7. GC-MS Analysis of Faecal Metabolites

**Figure 9:**
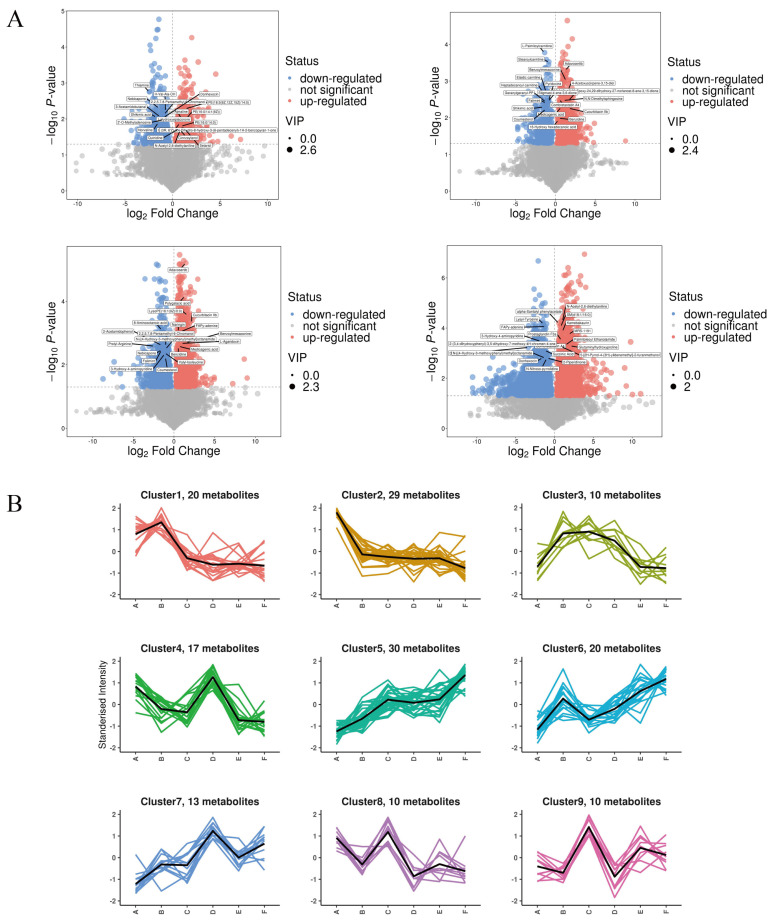
(**A**) Volcanic map of differential metabolites; (**B**) K-means clustering analysis of the differentially accumulated metabolites into nine clusters according to their expression profile. The cluster names and the number of metabolites for each cluster are indicated. (The masked metabolite in A vs. C is (23S, 24S)-17,23-Epoxy-24,29-dihydroxy-27-norlanost-8-ene-3,15-dione; A vs. F is Combretastatin A4).

As shown in Figure 9A, a total of 1256 qualitatively different metabolites were observed in group A and group B. Among them, 598 substances were up-regulated, and the top five metabolites with larger VIP values were PE (18:3(9Z,12Z,15Z)/14:0), Medicagenic acid, Setariol, and 20-Hydroxy-PGE2, in that order; a total of 658 substances were down-regulated, and the top five metabolites with larger VIP values were Shikimic acid, 2,6-Dimethylaniline, Thiamine, Styrene, and LysoPE (18:2(9Z,12Z)0:0), in that order. The q-con group compared with the PV group had a total of 1829 qualitatively different metabolites, of which 1107 were up-regulated, and the top five metabolites with larger VIP values were, in order, LysoPA (0:0/18:2(9Z,12Z), (23S,24S)-17,23-Epoxy-24,29-dihydroxy-27-norlanost-8-ene-3,15-dione, Benzoylmesaconine, Cucurbitacin IIb, and 4-Acetoxyscirpene-3,15-dio. A total of 722 substances were down-regulated in total, and the top five metabolites with higher VIP values were, in order, Heptadecanoyl carnitine, Oxytetracycline, Dethiobiotin, 2,6-Dimethylaniline, and (-)-Epigallocatechin. The total number of qualitatively different metabolites in group A and group E was 2112, of which 1058 were up-regulated, and the top five metabolites with larger VIP values were Palmitoylethanolamide, 4-Methyl-5-(-)-thiazoleethanol, and (-)-Epigallocatechin, in that order. Other metabolites include thiazoleethanol, Phenacetin, 2,3-Dinor-6-keto-prostaglandin F1 a, 2,4,6-(2,4,6-) trihydroxybenzoic acid, and 2,4,6-(2,4,6-) trihydroxybenzoic acid. A total of 1054 substances were down-regulated, and the top five metabolites with large VIP values were N-Acetylhistamine, Oxytetracycline, Etifoxine, Histamine, and MOPS, in that order.

To identify metabolites with consistent expression patterns in the clusters, we used the K-means clustering algorithm to group metabolites based on the similarity of metabolome profiles in Figure 9B. A total of nine clusters were identified, which could be divided into six categories: both A vs. C and D vs. E were up-regulated (categories 1 and 4); A vs. C was up-regulated but D vs. E was down-regulated (categories 2 and 7); A vs. C was up-regulated but D vs. E was unchanged (categories 3 and 5); both A vs. C and D vs. E were unchanged (category 6); both A vs. C and D vs. E were down-regulated (category 8); and A vs. C was down-regulated but D vs. E was unchanged (category 9). In addition, in the clusters with a general downward trend in metabolite accumulation, cluster 8 metabolite assembly was enhanced in A and C but decreased in D and E.

**Figure 10:**
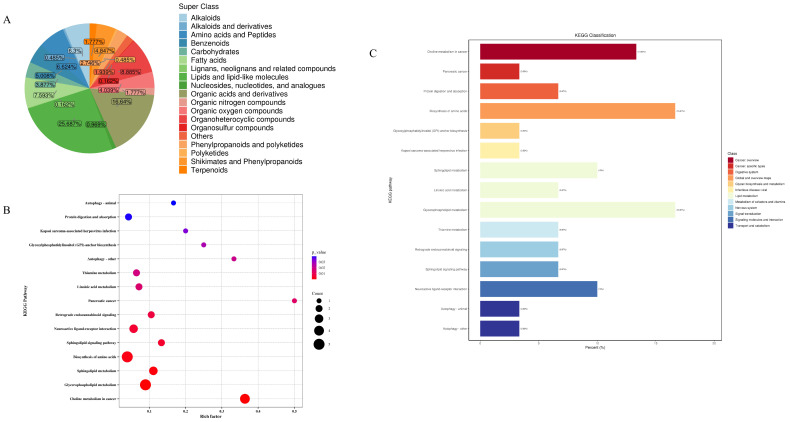
(**A**) Wayne diagram; (**B**) KEGG pathway analysis; (**C**) bubble map.

In this study, metabolomics analyses of mouse faecal samples from different groups were carried out, and the QC-TIC plots were superimposed on the multi-peak detection plots of the samples Figure 1, which demonstrated that the metabolomics data measured in the present study had good reproducibility and reliability. A total of 619 metabolites were identified, of which 159 (25.687%) were lipids and lipid-like molecules, 103 (16.64%) were organic acids and their derivatives, 55 (8.885%) were organic heterocyclic compounds, 47 (7.593%) were fatty acids (FA), 41 (6.624%) were amino acids and peptides, 39 (6.3%) were alkaloids, and benzene compounds had 31 species (5.008%), mangiferates and phenylpropanoates 30 species (4.847%), organic oxides 25 species (4.039%), carbohydrates 24 species (3.877%), phenylacetones and polyketides 17 species (2.746%), organic nitrogen compounds 11 species (1.777%), terpenoids 11 species (1.777%), nucleosides, nucleotides, and analogues 6 (0.969%), Alkaloids and derivatives 3 (0.485%), polyketides 3 (0.485%), lignans, neolignans, and related compounds 1 (0.162%), and organosulfur compounds 1 (0.162%) (Figure 10A). As shown in the high-level Wayne diagram, faecal metabolites of mice gavaged with different samples were both common and unique. The metabolic sets and pathways of 108 differential metabolites were analysed, and the results are shown in Figure 10B, where LWP affected 15 metabolic sets and no significant enrichment was observed. In Figure 10C, LWP affected fifteen pathways, of which four were significantly enriched: choline metabolism in cancer, glycerophospholipid metabolism, sphingolipid metabolism, and the biosynthesis of amino acids.

## 4. Discussion

Walnuts are a type of nut rich in unsaturated fatty acids [43], which has the efficacy of warming the lungs and fixing asthma, is a laxative, and promotes glucose utilisation, cholesterol metabolism, and cardiovascular protection [44,45]. Walnuts are mostly used for oil extraction, and the by-product walnut meal is rich in proteins. The extraction of walnut protein from walnut meal is an important direction for the development of the walnut industry in the future so as to realise the comprehensive utilisation of walnut meal, improve the added value of walnut kernel by-products, and reduce the eutrophication pollution of the environment. It has been found that walnut protein peptides prepared using different enzymes possess different physiological activities, including brain-health benefits, antioxidant properties, anti-cancer effects, anti-ageing effects, promotion of blood circulation, enhancement of the digestive system, prevention of radiation, insomnia relief, and improvement of endocrine system function [46,47,48,49]. This study used WP enzymatically digested by the oncidium protease, discovered by our team, to isolate LWPs with a molecular weight of less than 3000 Da. After gavage of LWP to the mice, the serum levels of BUN, LD, CK, and LDH of the mice were significantly elevated; in addition, the high-intensity exercise significantly reduced the food intake, HG, and MG of the mice but the experimental group that had been intervened by LWP had a relatively small reduction in HG and MG. This indicates that our prepared LWP has a certain alleviating effect on exercise fatigue in mice. The results of HE staining showed that LWP did not have any toxicological effect on mice and, in addition, the LWP intervention had a protective effect on skeletal muscle, reducing the damage to skeletal muscle cells caused by exercise and enhancing the exercise capacity of mice. In a study of macadamia nut hydrolysates, it was found that macadamia nut protein hydrolysates significantly prolonged weight-bearing swimming time, promoted hepatic glycogen synthesis, and reduced blood urea nitrogen and lactate in mice [50]; however, its anti-fatigue effect was not as excellent as that of LWP.

Separation and purification is a complex and time-consuming process, and peptide sequences that may be potentially biologically active can be screened quickly, efficiently, and cost-effectively by ultrafiltration separation, which can also reveal the mechanism of LWP activity at the molecular structure level [51]. A total of 4927 peptide sequences were identified in this study, and these peptides mainly consisted of 7–20 amino acids, with a predominance of short peptides with a molecular mass less than 1500 Da (61%). The subcellular localisation of differentially expressed proteins to the extracellular, nucleus, and cytoplasm was predominant, which was largely consistent with the results that cellular components were enriched in the extracellular region and extracellular vacuoles and were mainly enriched in the cytoplasm and nucleus. Among the biological functions mainly performed by LWP, the differential expression lies in the redox enzyme activity and molecular metabolism, and has a significant impact on the cellular and intracellular components. In the COG functional classification, the most frequently occurring proteins were cellular process and signal transduction proteins, and the least frequently occurring proteins were information storage and processing proteins. These results correspond to data from animal experiments.

The maintenance of gut microbial community diversity is very important for our life activities. In this study, the structural composition of gut microbes in mice gavaged with LWP was analysed using bacterial 16S rRNA high-throughput sequencing, and the results showed that there were no significant differences in the Ace, Chao1, Shannon, and Simpson indices of gut microbes in all groups. The composition of the mouse intestinal microbial community was mainly composed of four groups, *Firmicutes*, *Verrucomicrobiota*, *Bacteroidota*, and *Desulfobacterota*, which was the same as in previous studies. *Thick-walled bacterial phylum* is the most dominant group of bacteria in the intestine, which can help in the breakdown of complex carbohydrates, fatty acids, and polysaccharides in the intestine, and the higher relative abundance of *Thick-walled bacterial phylum* in the intestine may be related to the food source of mice. In this study, the positive control mice were gavaged with Vc and the experimental mice were gavaged with LWP, and these two gavages may have influenced the relative abundance of *Thick-walled phyla* in the intestinal microbial community of the mice. In 2004, researchers at the Microbiology Laboratory in Wageningen, The Netherlands, isolated *Akkermansia* from the faeces of healthy adults, and AKK has shown remarkable potential and advantages in the prevention and treatment of cardiovascular disease, colorectal cancer, and gouty arthritis. Although AKK bacteria account for only 3–5% of the intestinal flora, they are strongly associated with disease and health, and have been described as the “longevity bacterium” or the “strongest fighter among probiotics” [52]. *Muribaculum* is a major mucin-monosaccharide foraging bacterium that prevents the colonisation of *Clostridium* difficile while maintaining homeostasis in the intestinal tract. *Alistipes* is a Gram-negative bacterium in the phylum Mycobacterium, a relatively new genus of bacteria with protective effects against certain diseases, including liver fibrosis, cancer immunotherapy, and cardiovascular disease [53]. *Eubacterium siraeum* is a bacterium associated with adiposity and fatigue but has been linked to disease and health. *Siraeum* is a strain associated with fat deposition, which is effectively inhibited mainly by inhibiting the PI3K/AKT signalling pathway, which is a key regulator of lipid metabolism. In this study, we found that *Akkermansia*, *Muribaculum*, *Alistipes*, and *Eubacterium siraeum* were significantly increased in the LWP group, suggesting that consumption of LWP may be beneficial for long-term health.

In this study, we analysed the effect of LWP on faecal metabolic profiles using LC/MS. We identified 108 differential metabolites and four significantly enriched pathways. Palmitoylethanolamide, a natural human endogenous nutrient, has shown promising potential in relieving various pains, with anti-inflammation and neuroprotection properties [54], while 4-Methyl-5-thiazoleethanol is a common aroma ingredient in gastronomy, an ingredient with a brothy, nutty, and strong odour naturally found in beer, cocoa, and citrus fruits. In metabolite analyses of LWP-H mice, we found significant up-regulation of both Palmitoylethanolamide and 4-Methyl-5-thiazoleethanol. As outlined by the KEGG pathway enrichment analysis, LWP was significantly enriched in glycerophospholipid metabolism, biosynthesis of amino acids, and choline metabolism in cancer, and the metabolism of the related diseases may all be related to the degradation of LWP. LWP regulates a variety of physiological processes by affecting the metabolism of small molecule compounds but its regulatory mechanism needs further investigation. To determine its generalizability, the anti-fatigue activity can also be subsequently validated using other animal models or clinical trials. Therefore, LWP is not only easy to prepare and economical but also has good anti-fatigue effects, and its use in natural dietary supplements can add a unique flavour. In the future, it can be used to produce health food for fitness personnel, athletes, or people prone to fatigue.

## 5. Conclusions

In this study, we used Dregea sinensis Hemsl. Protease to enzymatically digest WP to obtain WPHs, identified as LWP after ultrafiltration, and then investigated its anti-fatigue effect and its effect on the intestinal microbiology and metabolic profiles of mice by gavaging mice with different doses of LWP. The experimental results showed that WPHs are rich in glutamic acid, which is an amino acid that can improve exercise fatigue. In the characterisation of LWP, differential protein expression was found to be concentrated in antioxidant-related pathways. The gavage of mice using LWP for 28 days revealed that LWP alleviated the rate of weight gain and prolonged the duration of weight-bearing swimming. The mice showed a significant increase in glycogen storage in muscle and liver, a decrease in serum levels of BUN, LDH, LA, and CK, an increase in levels of SOD and GSH, and a decrease in levels of MDA, predicting that the anti-fatigue efficacy of LWP is related to its ability to elevate oxidative stress in mice. In addition, LWP could increase the number of dominant strains, such as *Akkermansia*—a strain that enhances immunity and, thus, boosts anti-fatigue efficacy; furthermore, LWP restructured the gut microbiota by increasing the abundance of *Alistipes*, *Eubacterium*, and *Muribaculum*. Based on LC-MS metabolomics results, LWP was mainly enriched in glycerophospholipid metabolism and biosynthesis of amino acids, with notable metabolites including Palmitoylethanolamide and 4-Methyl-5-thiazoleethanol, all of which are beneficial to health. Therefore, LWP can delay the onset of fatigue to a greater extent and may be used as a natural dietary supplement due to its good efficacy and ease of extraction.

## Data Availability

The original contributions presented in the study are included in the article material, further inquiries can be directed to the corresponding author.
